# Delayed neurogenesis leads to altered specification of ventrotemporal retinal ganglion cells in albino mice

**DOI:** 10.1186/1749-8104-9-11

**Published:** 2014-05-18

**Authors:** Punita Bhansali, Ilana Rayport, Alexandra Rebsam, Carol Mason

**Affiliations:** 1 Department of Pathology and Cell Biology, College of Physicians and Surgeons, Columbia University, New York, USA; 2 Department of Neuroscience, College of Physicians and Surgeons, Columbia University, New York, USA; 3 Department of Ophthalmology, College of Physicians and Surgeons, Columbia University, New York, USA; 4 Current address: Department of Ophthalmology, Albert Einstein College of Medicine, New York, USA; 5 Current address: INSERM UMR-S839, Institut du Fer à Moulin, Paris, France

**Keywords:** Retinal ganglion cell, Albino, Neurogenesis, Cell specification, Zic2, Ipsilateral, Contralateral, Binocular visual pathway

## Abstract

**Background:**

Proper binocular vision depends on the routing at the optic chiasm of the correct proportion of retinal ganglion cell (RGC) axons that project to the same (ipsilateral) and opposite (contralateral) side of the brain. The ipsilateral RGC projection is reduced in mammals with albinism, a congenital disorder characterized by deficient pigmentation in the skin, hair, and eyes. Compared to the pigmented embryonic mouse retina, the albino embryonic mouse retina has fewer RGCs that express the zinc-finger transcription factor, Zic2, which is transiently expressed by RGCs fated to project ipsilaterally. Here, using Zic2 as a marker of ipsilateral RGCs, Islet2 as a marker of contralateral RGCs, and birthdating, we investigate spatiotemporal dynamics of RGC production as they relate to the phenotype of diminished ipsilateral RGC number in the albino retina.

**Results:**

At embryonic day (E)15.5, fewer Zic2-positive (Zic2^+^) RGCs are found in the albino ventrotemporal (VT) retina compared with the pigmented VT retina, as we previously reported. However, the reduction in Zic2^+^ RGCs in the albino is not accompanied by a compensatory increase in Zic2-negative (Zic2^−^) RGCs, resulting in fewer RGCs in the VT retina at this time point. At E17.5, however, the number of RGCs in the VT region is similar in pigmented and albino retinae, implicating a shift in the timing of RGC production in the albino. Short-term birthdating assays reveal a delay in RGC production in the albino VT retina between E13 and E15. Specifically, fewer Zic2^+^ RGCs are born at E13 and more Zic2^−^ RGCs are born at E15. Consistent with an increase in the production of Zic2^−^ RGCs born at later ages, more RGCs at E17.5 express the contralateral marker, Islet2, in the albino VT retina compared with the pigmented retina.

**Conclusions:**

A delay in neurogenesis in the albino retina is linked to the alteration of RGC subtype specification and consequently leads to the reduced ipsilateral projection that characterizes albinism.

## Background

Albinism is a genetic disorder associated with a disruption of melanin synthesis, resulting in the loss of pigmentation in the eyes, hair and skin or eyes only. In the eyes, the perturbation of melanogenesis is specifically in the retinal pigment epithelium (RPE), where it relates to numerous abnormalities of the visual pathways [1,2]. These abnormalities include defects in optic development and function, including impaired binocular vision [3-7]. Retinal ganglion cells (RGCs) establish the connections for binocular vision by projecting their axons either to the same (ipsilateral) or the opposite (contralateral) side of the brain. This ensures that higher visual centers receive information from both eyes to implement depth perception. In humans, 40% of RGCs project ipsilaterally, whereas in mice, only 3 to 5% of RGCs project ipsilaterally [8]. A hallmark of all albino mammals is a diminished ipsilateral projection [3,7,9].

In mice, contralateral RGC production begins in the center of the retina at embryonic day (E)11.5 and continuously expands to the periphery until postnatal day (P)0. Ipsilateral RGCs are produced during a shorter time span, approximately E11.5 to E16.5 [10], and specifically in the ventrotemporal (VT) region of the retina [8]. In addition, a population of RGCs in the VT retina that are born during late embryogenesis (approximately E15.5 to P0) projects contralaterally [10-12].

The pathway choice of an RGC axon at the optic chiasm is dependent on transcription factors that direct the expression of guidance receptors. The zinc-finger transcription factor Zic2 is expressed transiently in VT RGCs that project ipsilaterally between E14.5 and E17.5 and is critical for the formation of this projection [13]. The transcription factor Islet2 is expressed in contralateral RGCs (approximately 30% throughout the retina), but regulates only the late born crossed RGC projection from VT retina [14]. In albino mice, fewer RGCs express Zic2 during embryonic development [13], reflecting the reduction of the ipsilateral projection. The underlying mechanisms that cause the reduction of Zic2-expressing cells, however, are poorly understood.

The timing of melanin formation in pigmented mice coincides with the onset of RGC production [10,15,16]. Defects in melanin biogenesis in albinism have thus been thought to influence the pace of cell production, and consequently the specification of ipsilateral RGCs [17,18]. However, this relationship has not been directly demonstrated. Previous studies have described disruptions in the pace of RGC genesis in the albino retina, including a temporal lag in the differentiation of RGCs in the embryonic albino rat compared with the timing of RGC differentiation in the pigmented rat [19,20]. In contrast, we previously reported that more RGCs are produced at early stages in the albino retina than at early stages in the pigmented retina. We also demonstrated that, between E11 and E14, a greater percentage of ipsilateral RGCs are generated in the albino mouse retina than in the pigmented retina [21]. These studies, however, did not directly chronicle the timing of RGC neurogenesis in the albino mouse retina by using markers of RGC subtype projection.

Here, we report novel aspects of RGC production in the albino retina that influence ipsilateral and contralateral RGC specification. We capitalize on the transient expression of Zic2 in VT RGCs fated to project ipsilaterally and use EdU (5-ethynyl-2′-deoxyuridine) incorporation as a birthdating indicator to characterize the spatial distribution of ipsilateral RGCs and the timing of their generation in the albino and pigmented mouse retina. We show that, at E15.5, the reduction of Zic2-positive (Zic2^+^) RGCs is not accompanied by a compensatory increase in Zic2-negative (Zic2^−^) RGCs, resulting in fewer RGCs in the albino VT retina at this time. At E17.5, however, RGC number, including both Zic2^+^ and Zic2^−^ cells, becomes similar in the pigmented and the albino retina. Consistent with these findings, we show that the timing of RGC production is altered, such that fewer Zic2^+^ RGCs are born in the albino VT retina at earlier embryonic ages and more Zic2^−^ cells are produced at later embryonic ages. In addition, at this later stage, more VT RGCs express the contralateral marker Islet2 in albino retinae than in pigmented retinae. These results suggest that an alteration in the timing of production, and consequently specification of RGC subtypes, could explain the imbalance of ipsilaterally and contralaterally projecting RGCs in the albino visual system.

## Results

### The density of Zic2^+^ cells, but not their distribution, is changed at embryonic day 15.5 in the albino retina

**Figure 1:**
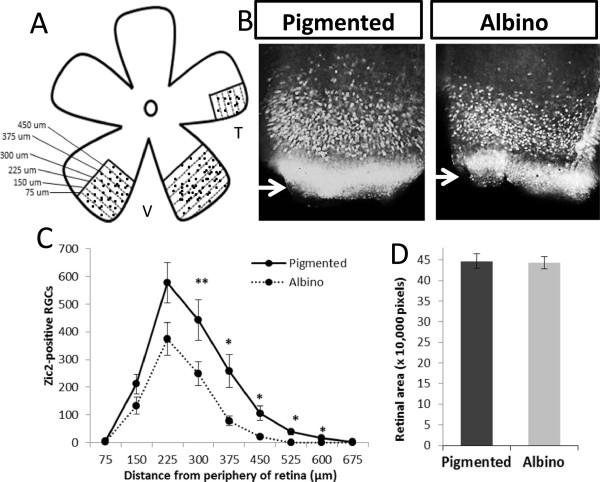
**The density of Zic2**^**+ **^**retinal ganglion cells, but not their distribution, is changed at embryonic day 15.5 in the albino retina. (A)** Cartoon depicting the method for counting Zic2^+^ retinal ganglion cells (RGCs) in 75 μm sectors from the periphery towards the center of the ventrotemporal (VT) retina. V, ventral; T, temporal. Black dots represent Zic2^+^ RGCs and dotted lines represent delineations of sectors. **(B)** Fluorescent images of retinal flat mounts immunostained with a Zic2 antibody at embryonic day 15.5. Zic2 is expressed in VT RGCs. **(C)** The number of Zic2^+^ cells in each sector of pigmented (n = 6) and albino (n = 6) VT retinae. In each sector, the number of Zic2^+^ cells is lower in albino compared to pigmented retina. However, the peak and distribution of Zic2^+^ cells are similar in the pigmented and albino retina. **(D)** The area of retinal flatmount does not differ in the pigmented (n = 11) and albino (n = 9) eyes at embryonic day 15.5. For area measurements, 1 pixel = 4.2734 μm^2^. Error bars indicate SEM. Two-tailed unpaired t-test: *P < 0.05, **P < 0.01.

Zic2 is transiently expressed in VT RGCs during development and regulates the ipsilateral trajectory [13,22]. We previously reported that fewer RGCs in the albino VT retina compared with the pigmented VT retina express the transcription factor Zic2 during the peak of Zic2 expression (E14.5 to E16.5) [13]. However, whether the reduction in Zic2^+^ RGCs in the albino retina reflects a smaller area of the VT retina that is occupied by Zic2^+^ RGCs or whether there is a decreased density of Zic2^+^ RGCs in the albino VT region has not been investigated. Using retrograde labeling of adult mouse RGCs by injection of horseradish peroxidase into the optic tract, Dräger and Olsen [23] previously reported that the area spanned by ipsilateral RGCs is not significantly different in albino and pigmented adult mice and that the decrease in the number of ipsilateral RGCs is apparent throughout the VT region. To further characterize the reduction of ipsilateral RGCs in the developing retina, we labeled ipsilateral RGCs using an antibody against Zic2 in pigmented and albino flat-mounted retinae (Figure 1B). Zic2^+^ RGCs were counted in 75 μm sectors from the periphery towards the center of the retina (Figure 1A). Zic2 is also expressed in cells of the ciliary marginal zone, a proliferative zone in the periphery of the retina, but these cells were not counted (Figure 1B, white arrows). At E15.5, the distribution and peak in the number of Zic2^+^ RGCs are similar in pigmented and albino retinae (Figure 1C). However, fewer Zic2^+^ RGCs are found in each sector of the albino retina at E15.5 compared with the pigmented retina (Figure 1C). This suggests that, in the albino retina, the density of Zic2^+^ RGCs is reduced throughout the entire VT region, but that the extent of the region containing Zic2^+^ RGCs is similar in pigmented and albino retinae. To discount the possibility that the reduction of Zic2^+^ RGCs is due to a reduction in the size of the retina, retinal areas were measured in pigmented and albino retinae and they were similar (Figure 1D), indicating that the decrease in Zic2^+^ RGCs in the albino is independent of the size of the retina.

Thus, these findings are in agreement with those of Dräger and Olsen [23] that the size of the region from which ipsilateral RGCs arise is similar in the albino and pigmented mouse retina, but that the density of ipsilateral RGCs is reduced. In addition, we show that the distribution of ipsilateral RGCs in the VT region of the albino retina is similar to that in the pigmented retina.

### At embryonic day 15.5, the albino ventrotemporal retina contains fewer postmitotic retinal ganglion cells

**Figure 2:**
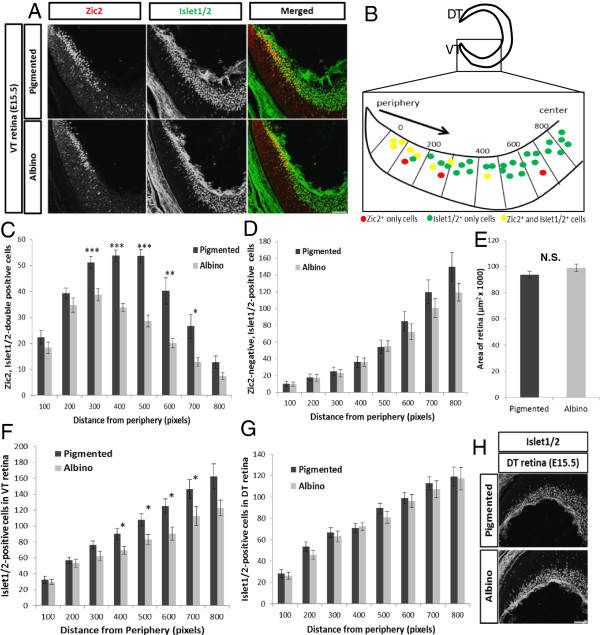
**The expression of Zic2 and Islet1/2 in the pigmented and albino retina at embryonic day 15.5. (A)** Frontal sections through ventrotemporal (VT) retina immunostained with antibodies against Islet1/2 (postmitotic retinal ganglion cells (RGCs), green) and Zic2 (ipsilateral RGCs, red) at embryonic day (E)15.5. Zic2^+^ RGCs are situated in the most peripheral region of VT retina. **(B)** (top) Cartoon depicting region of retina where Zic2^+^ RGCs reside in frontal sections. (bottom) Method of designating sectors from periphery to center for quantification and defining the VT region as the eight-most peripheral sectors of the retina. DT, dorsotemporal. All quantifications for (C-F) were carried out in two representative sections caudal to the optic nerve (see Methods). **(C)** At E15.5, fewer Zic2^+^ cells reside in the albino VT retina (n = 12) compared to the pigmented retina (n = 12) across sectors. **(D)** The number of Zic2^−^ RGCs is similar in pigmented and albino VT retinae. **(E)** The area of the retina in coronal sections (see Methods) is similar in pigmented (n = 7) and albino (n = 7) retina. **(F)** The number of Islet1/2^+^ cells is consequently reduced in albino VT retina (n = 12) across sectors compared with pigmented retina (n = 12). For distance from periphery, 1 pixel = 0.3205 μm. **(G)** Unlike in the VT retina, the number of Islet1/2^+^ cells in the DT retina at E15.5 is similar in pigmented (n = 7) and albino (n = 7) retina in each sector. Distance from periphery, 1 pixel = 0.3205 μm. **(H)** Islet1/2 expression in DT retina. No Zic2^+^ RGCs are present in DT retina. Error bars indicate SEM. Two-tailed unpaired t-test: *P < 0.05, **P < 0.01, ***P < 0.001. N.S., not significant. Scale bars in **(A)** and **(H)***,* 100 μm.

We next assessed whether the decrease in Zic2^+^ RGCs in the albino retina is accompanied by a concomitant increase in the number of Zic2^−^ RGCs. In coronal sections of the retina, we used an antibody against Islet1/2 in combination with the Zic2 antibody (Figure 2A). Islet1/2 serves as a marker for postmitotic RGCs during RGC development and other retinal cell types at later ages [24,25]. Two populations of cells, those that are Zic2^+^ and Islet1/2^+^ and those that are Zic2^−^ and Islet1/2^+^, were counted in radial sectors from the periphery to the center of the retina in representative sections of the retina (Figure 2B and see Methods).

In the albino retina at E15.5, there are fewer Zic2^+^ RGCs within a given sector (Figure 2C) and when summed across sectors (by 35.06%, *P* < 0.001). In contrast, the number of Zic2^−^ RGCs is similar in pigmented and albino VT retinae (Figure 2D), suggesting that only RGCs specified to express Zic2 are affected in the albino VT retina at this age. Because the reduction of Zic2^+^ RGCs is not accompanied by an increase in Zic2^−^ RGCs, the total number of Islet1/2^+^ (postmitotic) cells in the VT region of the albino retina is decreased in the more central sectors (Figure 2F) and when summed across sectors (reduced by 21.32%, *P* < 0.05). To discount the possibility that fewer RGCs are found in the albino retina due to differences in the size of the retina, the area of the retina was averaged in the same coronal sections used for counting RGCs and was similar in pigmented and albino mice (Figure 2E), consistent with area measurements in retinal whole mounts (Figure 1D).

To ascertain whether the reduction of Islet1/2^+^ cells is specific to the Zic2-expressing region in VT retina, Islet1/2^+^ cells in the dorsotemporal (DT) region of the retina (Figure 2H) were counted in the same representative sections used for VT analysis described above. Zic2 is not expressed in the dorsal retina. The number of Islet1/2^+^ cells in the DT retina was not significantly different in pigmented and albino retinae (Figure 2G). These results suggest that the decrease in RGCs at E15.5 is specific to Zic2^+^ RGCs and to the VT retina.

### At embryonic day 17.5, retinal ganglion cell number is similar in pigmented and albino ventrotemporal retinae

**Figure 3:**
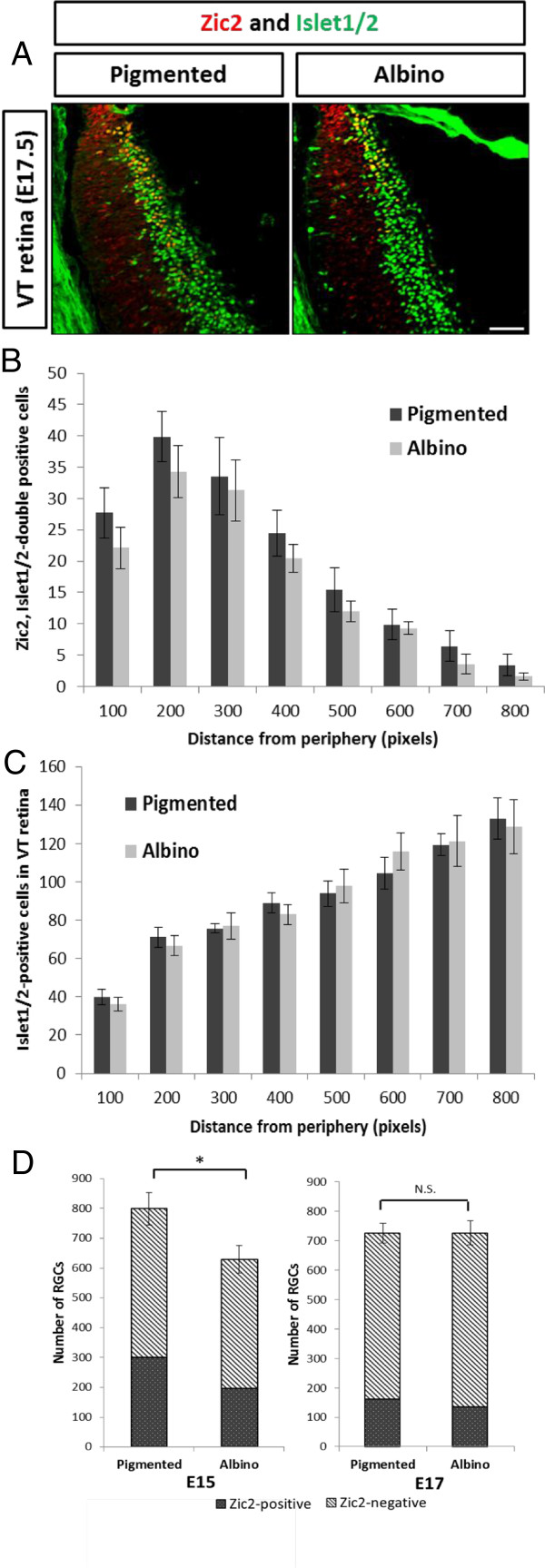
**The number of cells expressing Zic2 and Islet1/2 in the pigmented and albino ventrotemporal retina does not differ at embryonic day 17.5. (A)** Fluorescent sections through ventrotemporal (VT) retina stained with antibodies to Zic2 and Islet1/2 at embryonic day (E)17.5. Methodology of quantification in **(B)** and **(C)** is similar to the methods used in Figure 2, but representative sections are the second and fourth sections anterior to the optic nerve. **(B)** At E17.5, the number of Zic2^+^ cells is not significantly different in albino (n = 7) compared with pigmented (n = 7) retina in every sector. **(C)** The number of Islet1/2^+^ cells is also similar in the pigmented (n = 7) and albino (n = 7) retina at E17.5 across sectors. Distance from periphery: 1 pixel = 0.3205 μm. **(D)** Summary scheme of Figure 2 and Figure 3. At E15.5, the number of Zic2^+^ RGCs is reduced in the pigmented and albino, consequently diminishing the total RGC population. The number of Zic2^−^ RGCs is unchanged. At E17.5, the numbers of Zic2^+^ RGCs and Zic2^−^ RGCs are similar in pigmented and albino. Thus, the total number of RGCs is similar. Error bars indicate SEM. Two-tailed unpaired t-test: *P < 0.05. N.S., not significant. Scale bar in **(A)**, 100 μm.

Previous studies have shown that, at adult ages, the number of RGCs in the retinal periphery of pigmented and albino mice is approximately the same [23,26], suggesting that the reduction in RGCs that we observed at E15.5 does not persist to adulthood and that RGC numbers eventually equalize. To test this, we compared the numbers of Zic2^+^ and Islet1/2^+^ cells in VT retina of pigmented and albino mice at E17.5 (Figure 3A). Herrera and colleagues [13] showed that Zic2 is downregulated in RGCs after their axons have crossed the chiasm and expressed only in the most peripheral (immature) RGCs, implying that the majority of RGCs that express Zic2 at E15.5 do not express Zic2 at E17.5. In contrast to our observations in E15.5 retina, we found that the number of Zic2^+^ RGCs in the albino retina at E17.5 is not significantly different compared with the pigmented retina in all sectors, although there tend to be fewer Zic2^+^ RGCs in the albino retina (Figure 3B). Further, the number of Islet1/2^+^ cells in VT retina does not differ in pigmented and albino mice at E17.5 (Figure 3C). To confirm that all Islet1/2^+^ cells at E17.5 were RGCs as opposed to other cell types, as has been suggested in previous reports [24], Islet1/2 was co-immunostained with an antibody against Brn3, a marker of postmitotic RGCs [27]. All Islet1/2^+^ cells were found to be Brn3^+^ at E17.5 (data not shown).

In summary, the number of Zic2^+^ RGCs is reduced at E15.5 in albino retina compared with pigmented retina. Consequently, the total Islet1/2^+^ cell number (postmitotic RGCs) is reduced because there is no compensatory increase in Zic2^−^ RGCs (Figure 3D, left). At E17.5, however, the number of Zic2^+^ RGCs and the number of Islet1/2^+^ cells do not differ in pigmented and albino retina at E17.5 (Figure 3D, right).

### The timing of ventrotemporal retinal ganglion cell genesis is altered in albino retina

**Figure 4:**
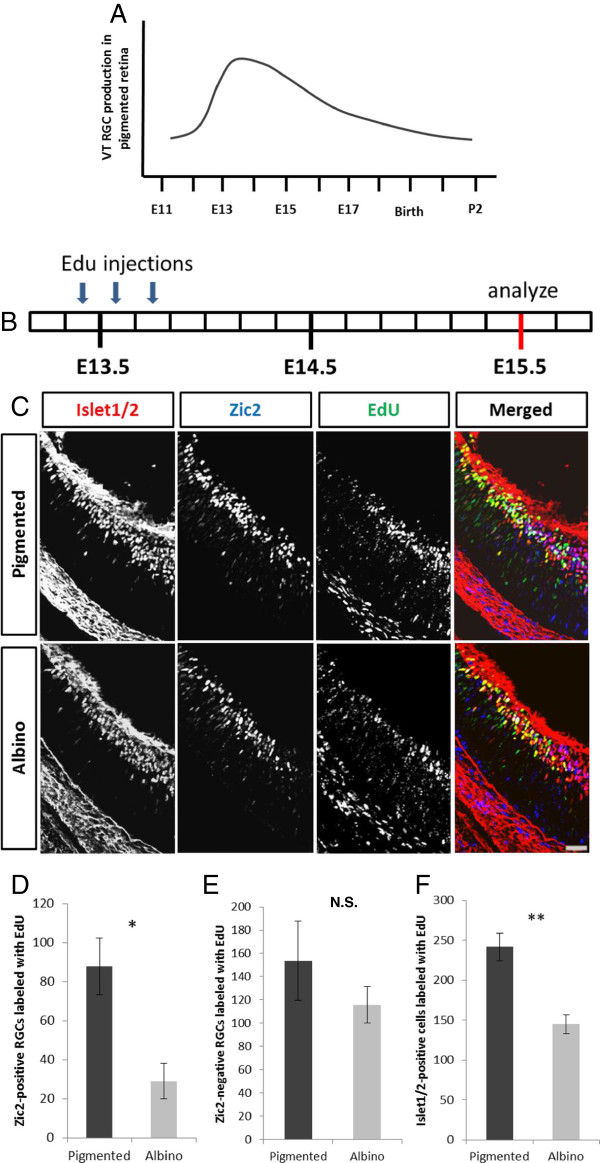
**Fewer Zic2**^**+ **^**retinal ganglion cells are born in albino than in pigmented ventrotemporal retina at embryonic day 13. (A)** Schematic of ventrotemporal (VT) retinal ganglion cell (RGC) production in pigmented retina (adapted from Young [29]). E, embryonic day; P, postnatal day. **(B)** Timeline of 5-ethynyl-2’-deoxyuridine (EdU) injections (10 am, 2 pm, 6 pm) at E13 and sacrifice at E15.5. Each box represents 4 hours. VT region defined as the most peripheral 800 pixels (1 pixel = 0.3205 μm)*.***(C)** Coronal sections of VT retina from pigmented and albino mice injected at E13 with EdU, collected at E15.5, and stained with antibodies to Zic2 and Islet1/2 and processed for EdU detection. All quantifications for **(D-F)** were carried out in the second and fourth representative sections caudal to the optic nerve. **(D)** Bar graph comparing the number of Zic2, Islet1/2-double positive cells in the VT region labeled with EdU in the pigmented retina and albino retina. Fewer cells expressing Zic2 and Islet1/2 at E15.5 become post-mitotic at E13 in albino (n = 4) compared with pigmented (n = 4) VT retina. **(E)** Bar graph comparing the number of Zic2^−^, Islet1/2^+^ cells in VT retina labeled with EdU in pigmented (n = 4) and albino (n = 4) retina. The number of Zic2^−^ RGCs produced at E13 is similar in pigmented (n = 4) and albino (n = 4) retina. **(F)** Bar graph comparing the number of total RGCs (Islet1/2^+^) labeled with Edu in pigmented (n = 4) and albino (n = 4) retinae. Significantly, fewer cells are Islet1/2^−^ and EdU^+^ in the albino retina. Error bars indicate SEM. Two-tailed unpaired t-test: *P < 0.05, ** *P* < 0.01. N.S., not significant. Scale bar in **(C)**, 50 μm.

**Figure 5:**
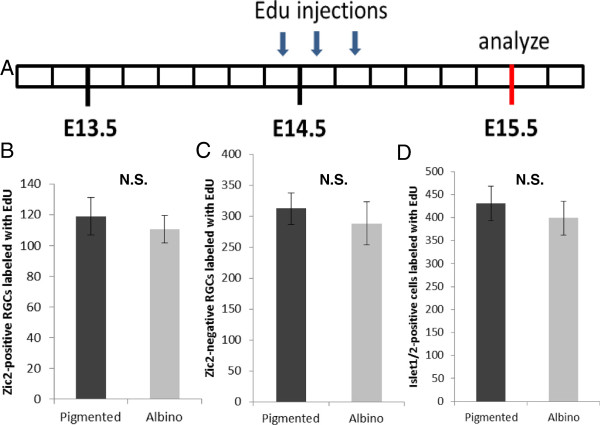
**A similar number of Zic2**^**+ **^**retinal ganglion cells are born in the pigmented and albino ventrotemporal retina at embryonic day 14. (A)** Timeline of 5-ethynyl-2’-deoxyuridine (EdU) injections (10 am, 2 pm, 6 pm) at embryonic day (E)14 and sacrifice at E15.5. Each box represents 4 hours. All quantifications for **(B-D)** were carried out in two alternating representative sections caudal to the optic nerve. Ventrotemporal (VT) region defined as most peripheral 800 pixels (1 pixel = 0.3205 μm)*.***(B)** Bar graph comparing the number of Zic2^+^ retinal ganglion cells (RGCs) in the VT region labeled with EdU in pigmented (n = 5) and albino (n = 5) retina. An equal number of Zic2^+^/Islet1/2^+^ cells become post-mitotic at E14.5 in albino VT retina compared with pigmented VT retina. **(C)** Bar graph comparing the number of Zic2^−^/Islet1/2^+^ cells in VT retina labeled with EdU in pigmented (n = 5) and albino (n = 5) retina. As in **B**, the number of Zic2^−^ RGCs at E15.5 that were born at E14 is similar in pigmented (n = 5) and albino (n = 5) retinae. **(D)** The number of total Islet1/2^+^ cells in VT retina labeled with EdU is also similar in pigmented and albino retinae. Error bars indicate SEM. Two-tailed unpaired t-test. N.S., not significant.

**Figure 6:**
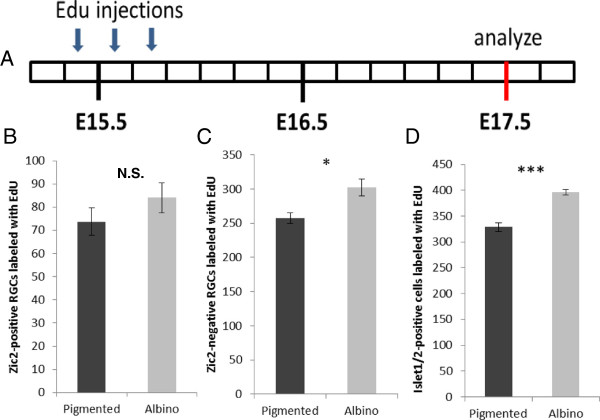
**More retinal ganglion cells are born in albino than in pigmented retina at embryonic day 15. (A)** Timeline of 5-ethynyl-2’-deoxyuridine (EdU) injections (10 am, 2 pm, 6 pm) at embryonic day (E)15 and sacrifice at E17.5. Each box represents 4 hours. All quantifications for **(B-D)** were carried out in the second and fourth sections rostral to the optic nerve. Ventrotemporal (VT) region defined as most peripheral 800 pixels (1 pixel = 0.3205 μm)*.***(B)** Bar graph comparing the number of Zic2^+^, Islet1/2^+^ cells in VT retina labeled with EdU in pigmented (n = 4) and albino (n = 4) retina. A similar number of Zic2^+^ RGCs are born at E15 in pigmented and albino retina. **(C)** Bar graph comparing the number of Zic2^-–^, Islet1/2^+^ cells labeled with EdU in pigmented (n = 4) and albino (n = 4) VT retina. A significantly greater number of Zic2^−^ RGCs are born at E15 in albino retina. **(D)** Bar graph comparing the number of Islet1/2^+^ cells in the VT region labeled with EdU in pigmented (n = 4) and albino (n = 4) retina. The number of Islet1/2^+^ cells at E17.5 that become post-mitotic at E15 is significantly higher in VT retina of albino mice (n = 4) compared with pigmented mice (n = 4). Error bars indicate SEM. Two-tailed unpaired t-test: * *P* < 0.05, ***P < 0.001. N.S., not significant.

The difference in the number of Islet1/2^+^ cells at E15.5 followed by an equalization of numbers at E17.5 in pigmented and albino VT retinae (Figure 3D) suggests a disparate pattern of RGC production in pigmented and albino VT retinae: fewer cells are produced before E15.5 and more cells are produced after E15.5 in the albino retina compared with the pigmented retina. Previous studies have described aberrations in the timing of cell genesis in the albino retina [19-21,28] but none have focused on the production of RGCs with respect to specification of projection subtype. To assess whether a difference in the time course of RGC production in the pigmented and albino VT retina is responsible for altered Zic2^+^ and Islet1/2^+^ cell numbers at E15.5 compared with E17.5, we performed a birthdating analysis using EdU incorporation in combination with Zic2 and Islet1/2 immunohistochemistry (IHC). In the pigmented mouse retina, the peak of RGC production occurs approximately between E13 and E16 (Figure 4A) [29]. To compare the production of VT RGCs expressing Zic2 in pigmented and albino retinae at E15.5, we injected pregnant mothers with EdU at E13 or E14, because after injections at these ages the number of brightly labeled cells is sufficient to provide a basis for comparison of albino versus pigmented neurogenesis. To compare the production of VT RGCs that express Zic2 at E17.5, we injected pregnant mothers with EdU at E15 or E16. However, none of the cells expressing Islet1/2 at E17.5 were labeled with EdU after injection at E16, suggesting that, at E16, the cell cycle is longer and cells require more than 1 day to become postmitotic (data not shown). Thus, the following three intervals were used between EdU injection and sacrifice: E13-E15.5, E14-E15.5, E15-E17.5 (Figures 4B, 5A and 6A).

After injections of EdU at E13 and sacrifice at E15.5 (Figure 4B,C), fewer Zic2^+^/EdU^+^ RGCs were found in the albino VT retina compared with the pigmented retina (Figure 4D) (reduction of 69.95%, *P* < 0.05), but the number of Zic2^−^/EdU^+^ RGCs was not significantly different (Figure 4E). These results suggest that fewer Zic2-expressing cells observed at E15.5 are born at E13 in the albino retina compared with the pigmented retina, while a similar number of RGCs that do not express Zic2 at E15.5 are born at E13. Accordingly, the number of Islet1/2^+^/EdU^+^ cells in this period was reduced in the albino retina compared with the pigmented retina (Figure 4F). The diminished production of Zic2^+^ RGCs at E13 in albino compared with pigmented retinae accounts for the reduction of Islet1/2^+^ cells present at E15.5 in the albino VT retina (Figure 2C,F).

Next, we compared pigmented and albino retinae in embryos at E15.5 whose mothers had received injections of EdU at E14 (Figure 5A). A similar number of Zic2^+^ and Zic2^−^ RGCs were labeled with EdU in pigmented and albino VT retinae (Figure 5B,C). This suggests that, at E14, the same number of RGCs are generated in the VT retina of pigmented and albino mice. Moreover, our results show that the reduction in RGCs that express Zic2 at E15.5 is the result of decreased cell production within the VT retina at E13, but not at E14.

If there are fewer RGCs in the albino VT retina at E15.5, but an equal number of RGCs at E17.5, as described in Figure 3D, then an increased rate of RGC production in the VT retina must occur in albinos between E15.5 and E17.5. To test this, we injected pregnant females with EdU at E15 and collected embryos at E17.5 (Figure 6A). The number of Islet1/2^+^ cells labeled with EdU at E17.5 was indeed greater in albino VT retinae than in pigmented VT retinae by 20.46% (Figure 6D). The number of Zic2^+^ RGCs at E17.5 that were born at E15 is not significantly different in pigmented and albino retinae (Figure 6B), whereas the number of Zic2^−^ cells that were labeled with EdU was greater in the albino VT retina compared with the pigmented retina (Figure 6C) by 17.40%. Thus, there are additional cells born in the albino VT retina at E15 compared with the pigmented retina, and these additional cells do not express Zic2.

Taken together, our findings indicate that the timing of cell production from E13 to E15 differs in pigmented and albino VT retinae. At E13 in the albino retina, fewer Zic2^+^ RGCs are produced in the VT retina compared with the pigmented retina, accounting for the reduction of Zic2^+^ RGCs and, thus, Islet1/2^+^ cells. At E14, the number of RGCs (both Zic2^+^ and Zic2^−^) produced in VT retina does not differ in pigmented and albino mice. At E15, however, more Islet1/2^+^ cells (specifically Zic2^−^) are produced in the albino retina than in the pigmented VT retina, leading to the equalization of the number of Islet1/2^+^ cells in pigmented and albino mice at E17.5 (Figure 3C). These results point to a delay in the genesis of VT RGCs in the albino retina.

### At embryonic day 17.5 more retinal ganglion cells express Islet2 in the albino ventrotemporal retina compared with pigmented retina

**Figure 7:**
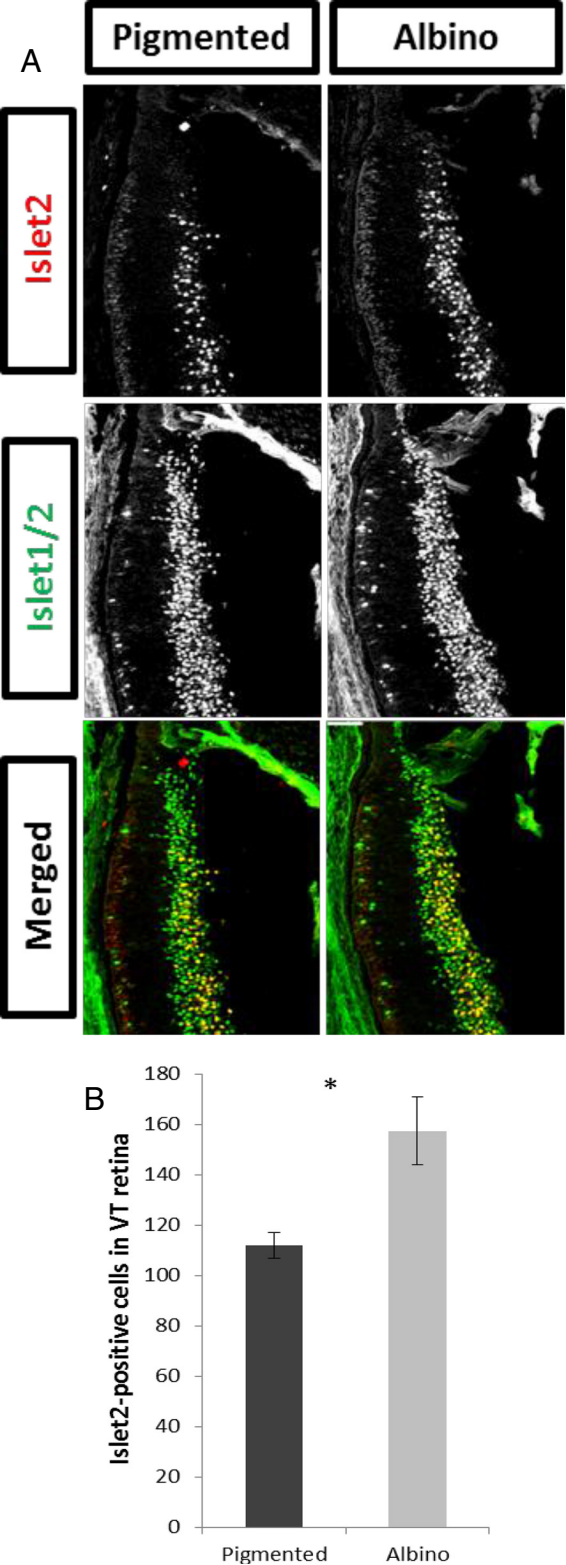
**More Islet2**^**+ **^**retinal ganglion cells are found in albino compared with pigmented ventrotemporal retina at embryonic day 17.5*. *(A)** Islet2 (red) and Islet1/2 (green) expression as revealed by immunohistochemistry in the pigmented and the albino ventrotemporal (VT) retina at embryonic day 17.5. **(B)** The number of Islet2^+^ retinal ganglion cells is greater in the albino (n = 4) VT retina compared with the pigmented (n = 4) VT retina (*P* = 0.0361). Error bars indicate SEM. Two-tailed unpaired t-test: *P < 0.05. Scale bar in **(A)** bottom right panel, 100 μm.

The quantification of cells expressing Zic2 and Islet1/2 in combination with EdU birthdating indicates that more RGCs are born at E15 in the VT retina of albino mice compared with pigmented mice. Since these additional late-born RGCs do not express Zic2, we hypothesized that they may be fated to project contralaterally. Anterograde tracing data described by Rebsam and colleagues [30] showed that a cohort of contralateral fibers from the albino VT retina forms an aberrant segregated patch in the dorsal lateral geniculate nucleus (dLGN) near the dorsal tip, the area innervated by the late-born crossing fibers. These data point to the possibility that a population of cells in the VT retina that would have been fated to project ipsilaterally, as in the pigmented mouse, is instead mis-specified to project contralaterally in the albino mouse. The transcription factor Islet2 is expressed in the RGCs of non-VT retina during the peak of Zic2 expression and then expands its expression into VT retina from E17.5 to postnatal ages [14]. Islet2 is expressed in approximately 30% of all crossing RGCs but appears to selectively regulate the late-crossing VT population [14]. Previously, our qualitative assessment indicated that Islet2 expression is not altered in the albino retina [30]. However, because our EdU experiments revealed an increase in Zic2^−^ RGC neurogenesis at E15, we hypothesized that these cells might express contralateral markers. To test this, we quantitatively compared Islet2 expression in pigmented and albino retinae at E17.5 and found that the number of Islet2^+^ RGCs in albino VT retinae is greater than in pigmented retinae (Figure 7A,B). This result supports our hypothesis that more cells are specified to project contralaterally at E17.5 and that in albino mice, as shown in other albino higher vertebrates, the reduction of the ipsilateral projection is likely accompanied by an increase in the contralateral projection.

## Discussion

Our analyses demonstrate perturbations in the genesis of RGCs in the VT region of the albino retina that can be linked to the aberrant development of the binocular visual pathway characteristic of albino mammals. We take advantage of the expression of transcription factors, Zic2 and Islet2, which correlate in space and time with the development of RGCs that project ipsilaterally and contralaterally, respectively. Altered cell production in the albino retina may affect RGC specification, in turn resulting in a diminished ipsilateral projection [3,4] and mistargeting in the dLGN [30].

### Retinal ganglion cell number is reduced in the albino retina in a region- and time-specific manner

Compared with the pigmented retina, fewer Zic2^+^ RGCs are found in the albino VT retina at E15.5. This decrease is not compensated by an increase in Zic2^−^ RGCs, and thus the total number of Islet1/2^+^ (postmitotic) RGCs in the peripheral VT retina is reduced in the albino mouse at this time. A reduction in RGC number in the central retina at postnatal ages has been described previously in non-rodent albino species [26,31]. Moreover, some albino species exhibit a decrease in other cell types throughout all layers of the central retina [26,31,32] and a rod deficit [33,34]. Our study is the first to describe a transient reduction in RGC number in the peripheral VT region, from which the ipsilateral RGCs originate, and during embryonic ages. The reduction in Zic2^+^ RGCs, but not Zic2^−^ RGCs, suggests that Zic2^+^ RGCs are differentially affected in the albino mouse retina. In addition, we did not find a similar reduction in RGC number in the DT retina of albinos at E15.5, suggesting that the transient decrease in RGC number is specific to the VT retina and Zic2^+^ RGCs. These findings suggest that the VT retina is intrinsically different from the regions that produce only contralateral RGCs.

In our previous study [21], we compared the number of Islet1/2^+^ cells in pigmented and albino mice at embryonic ages in retinal whole mounts, but did not observe a change in total RGC counts. Nonetheless, we noted a thinning of the RGC layer in the ventral pole of the albino retina at E14 that is more pronounced compared with that of the pigmented retina. This thinned area could reflect the diminished number of RGCs in the VT retina that we observed in the present study. We also compared the total number of Islet1/2^+^ cells in flat-mounted retina over a large area, in which subtle differences in cell number could have been diluted [21]. In contrast, the present experiments quantified Islet1/2-expressing cells in sections through more specific and smaller regions of albino and pigmented retinae.

### The wave of ventrotemporal retinal ganglion cell genesis is delayed in the albino

**Figure 8:**
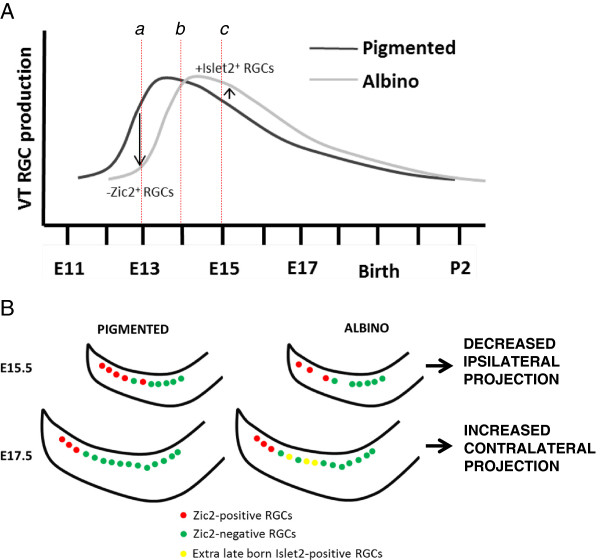
**A delay in ventrotemporal retinal ganglion cell production during embryogenesis alters the proportion of ipsilateral and contralateral retinal ganglion cells. (A)** Scheme of the wave of ventrotemporal (VT) retinal ganglion cell (RGC) production in the albino and pigmented retina and its link to RGC projection subtype specification. (a) At embryonic day (E)13, fewer VT RGCs, and specifically Zic2^+^ RGCs, are produced in the albino retina compared with pigmented retina. (b) At E14, a similar number of VT RGCs, both those that are Zic2^+^ and those that are Zic2^−^, are produced in pigmented and albino retinae. (c) At E15, more RGCs, specifically Zic2^−^ RGCs, are produced in the albino retina compared with the pigmented retina. The additional late-born Zic2^−^ RGCs express contralateral markers, reflected in an increased number of Islet2^+^ RGCs in the albino VT retina compared with the pigmented VT retina. P, postnatal day. **(B)** Scheme depicting differences in Zic2^+^ and Islet1/2^+^ cell numbers in the albino retina resulting from the delay in VT RGC production described in **(A)**. The delay alters the number of cells expressing Zic2 (red) at earlier ages (E15.5) to produce fewer ipsilateral RGCs and the number of cells expressing Islet2 at later ages (E17.5) to produce an extra population of contralaterally projecting RGCs (yellow). The number of overall VT RGCs in the albino mouse equalizes at E17.5.

We used EdU birthdating in combination with charting Zic2 and Islet1/2 expression at the time of sacrifice. Fewer RGCs observed at E15.5 are produced at E13 in the albino retina compared with the pigmented retina (Figure 8Aa). A similar number of RGCs present at E15.5 are generated at E14 in pigmented and albino retinae (Figure 8Ab). One day later, however, at E15, a greater number of RGCs are born in the albino compared with the pigmented retina at E17.5 (Figure 8Ac). Taken together, these results point to a delay in the production of VT RGCs in the albino retina compared with the pigmented retina.

Our findings are consistent with studies that have described a temporal lag in the differentiation of RGCs in embryonic albino rats compared with pigmented rats [19,20]. The results presented here do not ostensibly agree with the data in [21] that the albino exhibits an early overproduction of ipsilateral RGCs. These discrepant findings can be attributed to differences in the methods of data collection and display: in our previous study [21], we used bromodeoxyuridine (BrdU) birthdating at embryonic ages in combination with retrograde labeling via the optic tract at postnatal ages, whereas in the present study, we utilized EdU birthdating in combination with Zic2 expression at embryonic ages to chart VT RGCs at the time when they are specified to project ipsilaterally. Moreover, in our previous study, we quantified ipsilateral RGCs born at each age as a percentage of total ipsilaterally projecting RGCs (BrdU^+^/dextran^+^) at postnatal ages, and reported an increase in early-born ipsilateral RGCs in the albino retina. However, as the total number of ipsilaterally projecting RGCs is reduced in the albino retina, such a calculation could bias this percentage to reflect an increase in production.

The shift in the wave of VT RGC genesis in the albino retina observed in the present study could occur as a result of altered cell cycle length and/or the failure of cells to exit the cell cycle at the proper time. Previous studies have reported that the number of cells undergoing mitosis postnatally in the albino rat is greater in regions where rod number is reduced [17,18], suggesting that precursors of other retinal cells may also fail to exit the cell cycle at the appropriate time. The possibility that the reduced number of RGCs in the albino VT retina during embryonic ages is accompanied by a greater number of proliferating cells is consistent with our previous finding that the temporal retina is expanded [21]. The expansion may reflect a population of cells in the albino retina that have failed to exit the cell cycle, leading to fewer postmitotic RGCs at early stages, that then become postmitotic later in development, as we document here. In addition to parameters of timing, other features of proliferation, such as mitotic spindle orientation, have been shown to influence cell differentiation and specification [35]. In postnatal albino rats, the proportion of mitotic spindles oriented vertically in the retina, associated with asymmetric cell division and maintenance of proliferation, is significantly higher than in pigmented rats [18]. To address whether the decrease in VT RGCs results from abnormal cell cycle parameters, a thorough analysis of cell cycle regulation is needed for the albino mouse retina.

### The timing of retinal ganglion cell production is related to subtype specification

Our results suggest that alterations in the rate of cell production are correlated with changes in cell specification: we show that the production of RGCs is delayed in the albino VT retina, that this delay is linked to the altered expression of genes that regulate ipsilateral and contralateral fate, and, consequently, that the decussation of RGC axons is perturbed. At E13, fewer Zic2^+^ RGCs, but not Zic2^−^ RGCs, are produced, implicating the delay in differentiation as specific to the Zic2^+^ cohort of Islet1/2^+^ cells. One explanation is that cells that express Zic2 and are fated to project ipsilaterally arise from a different set of progenitors than those fated to project contralaterally and that the Zic2^+^ progenitors are preferentially altered in the albino retina. Ipsilateral RGCs are produced during a shorter period than contralateral RGCs [10], further supporting the idea that ipsilateral RGCs arise from a different pool of progenitors. Our results thus suggest that only the pool of progenitors slated to express Zic2 is affected in the albino mouse, thereby reducing Zic2^+^ but not Zic2^−^ RGCs at early ages. Due to their delayed production, the additional RGCs born at E15 in the albino retina may have lost their competence to express Zic2, and instead express contralateral markers such as Islet2. Accordingly, we found that the number of Islet2^+^ RGCs at E17.5 is greater in the albino VT retina than in the pigmented retina, in contrast to our previous qualitative estimates that did not reveal a difference in Islet2 expression at E17.5 [30]. Thus, these extra late-born RGCs may correspond to the cohort of contralateral VT RGCs that target close to the region innervated by normal late-born contralateral fibers but abnormally cluster together in the dLGN [30].

The increased number of Islet2^+^ RGCs in the albino suggests that more cells are specified to project contralaterally in the albino mouse retina compared with the pigmented mouse retina. Concomitant with the smaller ipsilateral projection, an increase in contralateral fibers has been reported in albino ferrets, cats, and humans [36,37]. However, in rodents, where the proportion of contralateral RGCs is high (95 to 97%), a complementary increase has not been reported in the albino retina. In fact, Dräger and Olsen [23] estimated the number of contralateral RGCs in retrogradely labeled retinae by sampling regions throughout the retina and found no significant differences in the number of contralateral projections between mature albino and pigmented retinae. The present findings are at odds with our previous study [30], in which we qualitatively analyzed the pigmented and albino VT retina and found no difference in the extent of Islet2^+^ cells. Here, we instead precisely quantified Islet2^+^ RGCs in consistently selected sections and along a defined distance from the peripheral border of the VT retina (delineated by the most peripheral Islet1/2^+^ cells). This approach provides a more reliable assessment of the number of Islet2^+^ cells in pigmented and albino retina.

Different retinal cell classes proliferate and differentiate with stereotypic timing [38-41]. In species such as cat, ferret, monkey and rat, morphological (based on soma size) and functional (that is, X, Y, and W) classes of RGCs are born in a specific sequence [42-46]. The birth order of different RGC subtypes has also been related to chiasmatic crossing [10,44]. Our data suggest that, when the timing of neurogenesis is disrupted, ipsilateral and contralateral specification related to chiasmatic decussation is altered. Our findings are also in agreement with analyses of other brain regions and species illustrating that precise patterns of proliferation and neurogenesis regulate cell fate decisions, and that the length of cell cycle phases and time of birth can influence the proper proportion of different cell types [47-50]. Moreover, similar to our findings in the VT retina, it has been shown in the neocortex that variations in cell cycle length and transcription factor expression collaborate during the production of specific cell types and determination of their ultimate position [51].

## Conclusions

Our study provides further support for the relationship between the timing of neurogenesis and cell fate specification in the mammalian retina and elucidates a mechanism by which the ipsilateral projection is reduced in the albino retina.

We show a delay in the timing of RGC production in the albino mouse, specifically in the VT retina, that can be related to a decrease in the number of RGCs fated to project ipsilaterally (those that express Zic2) and an increase in the number of RGCs that express Islet2. The perturbation in RGC genesis may underlie the establishment of abnormal retinal axonal divergence in albinism.

## Methods

### Animals

Mice were obtained from The Jackson Laboratory (Bar Harbor, ME, USA) and housed in a timed-pregnancy breeding colony at Columbia University. Conditions and procedures were approved by the Columbia University Institutional Animal Care and Use Committee, protocol numbers AAAG8702 and AAAG9259. The albino model employed was the C57BL/6 J mouse that carries a single nucleotide mutation in the tyrosinase gene (Tyr^c2j^) and is thus coisogenic with pigmented controls other than the expression of tyrosinase. Heterozygote mice (Tyr^+/c2j^) were crossed with homozygote tyrosinase mutants (Tyr^c2j/c2j^), and their litters contained homozygous albino embryos and heterozygous pigmented embryos that we used as controls. Thus, “albino” and “pigmented” refer to homozygote Tyr^c2j/c2j^ and heterozygote Tyr^+/c2j^, respectively. Females were checked for vaginal plugs at approximately noon each day. E0.5 corresponds to the day when the vaginal plug was detected, with the assumption that conception took place at approximately midnight.

### Retinal whole mount immunohistochemistry

Embryos were harvested at E15.5, washed in PBS, decapitated, and heads were fixed by immersion in 4% paraformaldehyde in 0.1 M phosphate buffer overnight at 4°C. After the heads were rinsed in PBS several times, a small radial slit was made at the ventral pole of the retina for future orientation, the lens was removed, and the retina was dissected free from the eye lids and socket and detached from the RPE. The dissected retinae were blocked in 10% normal goat serum (NGS), 1% Triton in PBS for 1 to 2 hours at room temperature, incubated in primary antibody (Zic2, 1:10,000, gift of Stephen Brown, University of Vermont) in 1% NGS, 1% Triton in PBS overnight at 4°C, washed in PBS 3 × 20 minutes, incubated in secondary antibody (Donkey anti-rabbit Alexa488; InVitrogen Molecular Probes, Carlsbad, CA, USA; 1:400) in 1% NGS, 1% Triton in PBS for 2 hours at room temperature, and washed in PBS 3 × 20 minutes. The retinae were then flat-mounted on a Fisherbrand® Frosted slide (Pittsburgh, PA, USA), making 4 to 5 cuts at equidistant points along the periphery, each one in a radial direction from center to periphery, and then coverslipped with Fluoro-Gel (Electron Microscopy Sciences, Hatfield, PA, USA).

The region of the retina expressing Zic2 was imaged with a Zeiss Axioplan2 microscope (Zeiss, Thornwood, NY, USA) and 20x objective with an Axiophot camera and Axiovision software (Zeiss, Thornwood, NY, USA). For quantification of Zic2^+^ cells in retinal whole mounts, multiple 20x images of the Zic2-expressing region were stitched together to create a final image containing the entire Zic2 zone in the VT retina using Adobe Photoshop CS6 (Adobe Systems Incorporated, San Jose, CA, USA). Zic2^+^ RGCs were counted in sectors of equal width (75 μm) from the periphery to the center of the retina through the Zic2-expressing RGC territory, using ImageJ (National Institutes of Health, Bethesda, MD, USA) (Figure 1A). All counts were carried out in a semi-blinded manner (the pigment in the retinal choroidal melanocytes indicated genotype). The Zic2^+^ cells in the ciliary marginal zone, which are more densely packed and morphologically distinct compared to Zic2^+^ RGCs, were excluded from quantification. The area of flat mounted retina was measured using the Region Tracing Tool on the MetaImaging Series, MetaMorph 7.0 (Molecular Devices, Sunnyvale, CA, USA) (1 pixel = 4.2734 μm^2^).

### Immunohistochemistry of retinal sections

Embryos were collected at E15.5 and E17.5, heads were decapitated in PBS over ice, fixed in 4% paraformaldehyde in 0.1 M phosphate buffer for 1 to 2 hours at 4°C, rinsed in PBS at least three times, washed in PBS for a minimum of 1 hour at 4°C, and cryoprotected in 10% sucrose in 0.1 M PBS for 24 or 48 hours at 4°C. The heads were embedded in Tissue-Tek® optimal cutting temperature compound (Sakura Finitek USA, Torrance, CA, USA) in Peel-A-Way Disposable Embedding Molds (Polysciences, Inc., Warrington, PA, USA) over crushed dry ice and stored in a -80°C freezer until sectioned. Frontal sections (20 μm) were cut on a cryostat (Leica Biosystems, Buffalo Grove, IL, USA) and collected on Fisherbrand® Frosted slides. Sections were either stored in the -80°C freezer or used immediately for IHC. For IHC, slides were blocked in 10% NGS, 0.2% Triton in PBS for 1 hour, incubated in primary antibody in 0.2% Triton, 1% NGS, in PBS overnight at 4°C, washed 3 × 20 minutes in PBS at room temperature, incubated in secondary antibody for 2 hours at room temperature, and washed in PBS 3 × 20 minutes at room temperature. Slides were coverslipped with Fluoro-Gel.

The following primary antibodies were used: rabbit anti-Zic2 (gift of Stephen Brown, 1/10,000), mouse anti-Islet1/2 (1/50-1/100), rabbit anti-Islet2 (1:3,000-8,000). Both Islet antibodies were gifts of Susan Morton and Thomas Jessell, Columbia University. The Islet1/2 antibody was made against Islet1 (expressed in postmitotic RGCs) and also recognizes Islet2 (expressed in a subset of RGCs) [14,25]. The following secondary antibodies were used: Donkey anti-rabbit Alexa594 (InVitrogen Molecular Probes; 1:400), Goat anti-mouse Alexa488 (InVitrogen Molecular Probes; 1:400), Donkey anti-mouse Alexa594 (InVitrogen Molecular Probes; 1:400), Donkey anti-rabbit Alexa488 (InVitrogen Molecular Probes; 1:400), Goat anti-rabbit Cy5 (Jackson Immunoresearch, West Grove, PA, US; 1:250).

Stained sections of the retina were imaged using a Zeiss AxioImager M2 microscope equipped with ApoTome, AxioCam MRm camera, and Neurolucida software (V10.40, MicroBrightField Systems, Williston, VT, USA). A merged stack of eight images (2 μm steps) was acquired with the ApoTome and 20x objective.

All quantifications for sections processed for IHC were performed using Meta Imaging Series Metamorph 7.0. The inner surface of the retina was divided into radial sectors of equal width (100 pixels, 1 pixel = 0.3205 μm) from the periphery to the center (Figure 2B). For each sector, a line was drawn from the inner surface of the retina towards the outer surface in the axis of cell migration (Figure 2B). Cell numbers were recorded for each individual sector. The first sector was circumscribed by the peripheral-most border of Islet1/2^+^ cells. Images were thresholded with the Metamorph program such that only the most strongly labeled cells were counted. Retinal area was measured using the Region Tracing Tool on the Metamorph imaging system and 1 pixel = 1.64 μm^2^. For statistical analysis at E15.5, the second and fourth sections caudal to the section where the optic nerve is attached to the eye were chosen because the Zic2^+^ cells in pigmented retina are most numerous in these sections. The number of Islet1/2^+^ RGCs and the number of Zic2^+^ RGCs in these two sections were summed and then averaged across individuals. At E17.5, the first and third sections rostral to the section where the optic nerve exits the eye were chosen for quantitation, as the orientation of the retina with respect to the optic nerve has changed from E15.5 to E17.5 and these sections captured the Zic2 population. Student’s t-test was performed for all statistical analysis.

### 5-Ethynyl-2’-deoxyuridine labeling

EdU (2.5 mg/ml, InVitrogen) was injected into the pregnant mother intraperitoneally on the day of interest at roughly 10 am, 2 pm, and 6 pm. EdU, similar to BrdU and [^3^H]thymidine, is incorporated into DNA and labels cells undergoing S-phase [52]. Embryos were collected from pregnant females and sacrificed at E15.5 or E17.5. The embryos were prepared and processed for IHC as per the procedure described above for retinal sections. Upon completion of immunostaining for Zic2 and Islet1/2, the sections were permeabilized in 0.5% Triton in PBS for 20 minutes at room temperature, washed in PBS 2 × 5 minutes at room temperature, and the EdU detection reaction performed as per the protocol described in Salic and Mitchison [52], using a fluorescent azide through a Cu(I)-catalyzed cycloaddition reaction (Click-iT EdU imaging kit, Invitrogen, Carlsbad, CA, USA), for 30 minutes at room temperature. After EdU revelation, slides were washed 2 × 10 minutes in PBS at room temperature. Sections were coverslipped with Fluoro-Gel. Imaging and quantification were performed as described above. The EdU signal in retinae injected at E13 and collected at E15.5 was thresholded such that only the cells that became post-mitotic at E13 (brightest) were counted, using the Meta Imaging Series software Metamorph 7.0. The EdU signal in retinae injected at E14 and collected at E15.5 did not require thresholding because the window between the age of injection and collection was too short for multiple rounds of division, minimizing the disparity in EdU signal strength amongst postmitotic RGCs. The EdU signal in retinae injected at E15 and collected at E17.5 did not require thresholding, even though the time window between injections and collection was 2.5 days. In pregnant mothers injected with EdU at E16.5 and sacrificed at E17.5 for collection of embryos, no Islet1/2^+^ cells were labeled with EdU (data not shown), confirming that cell cycle is longer at later ages [41] and eliminating the need to threshold for the E15 injection-E17.5 collection time window.

## Abbreviations

BrdU: bromodeoxyuridine; dLGN: dorsal lateral geniculate nucleus; DT: dorsotemporal; E: embryonic day; EdU: 5-ethynyl-2’-deoxyuridine; IHC: immunohistochemistry; NGS: normal goat serum; P: postnatal day; PBS: phosphate buffered saline; RGC: retinal ganglion cell; RPE: retinal pigment epithelium; VT: ventrotemporal.

## Competing interests

The authors declare that they have no competing interests.

## Authors’ contributions

PB participated in the design and coordination of the study, carried out the experiments and statistical analysis, and drafted, wrote and edited the manuscript. IR assisted with design and execution of cell counts for Figure 1. AR helped to design the study and to write and edit the manuscript. CM conceived of and coordinated the study, and helped to draft, write, and edit the manuscript. All authors read and approved the final manuscript.
